# Endothelial cell activation and neovascularization are prominent in dermatomyositis

**DOI:** 10.1186/1740-2557-3-2

**Published:** 2006-02-20

**Authors:** Kanneboyina Nagaraju, Lisa G Rider, Chenguang Fan, Yi-Wen Chen, Megan Mitsak, Rashmi Rawat, Kathleen Patterson, Cecilia Grundtman, Frederick W Miller, Paul H Plotz, Eric Hoffman, Ingrid E Lundberg

**Affiliations:** 1 Children's National Medical Center, Research Center for Genetic Medicine, 111 Michigan Ave NW, Washington DC, 20010, USA; 2 Environmental Autoimmunity Group, NIEHS, National Institutes of Health, Department of Health and Human Services, Bethesda, MD, USA; 3 Johns Hopkins School of Medicine, Baltimore, MD, USA; 4 Arthritis and Rheumatism Branch, NIAMS, National Institutes of Health, Department of Health and Human Services, Bethesda, MD, USA; 5 Children's Hospital Medical Center, Seattle, WA, USA; 6 Rheumatology Unit, Department of Medicine, Karolinska University Hospital, Solna, Stockholm, Sweden

## Abstract

**Background:**

While vascular and immune abnormalities are common in juvenile and adult dermatomyositis (DM), the molecular changes that contribute to these abnormalities are not clear. Therefore, we investigated pathways that facilitate new blood vessel formation and dendritic cell migration in dermatomyositis.

**Methods:**

Muscle biopsies from subjects with DM (9 children and 6 adults) and non-myositis controls (6 children and 7 adults) were investigated by immunohistochemistry using antibodies that recognize existing (anti-CD146) and newly formed blood vessels (anti-αVβ3) and mature dendritic cells (anti-DC-LAMP). Blood vessel quantification was performed by digitalized image analysis. Additional muscle biopsies from subjects with adult DM and non-myositis controls were used for global gene expression profiling experiments.

**Results:**

A significant increase in neovascularization was found in muscle biopsies of DM patients; neovascularization (αVβ3 positive capillaries and vessels per muscle fiber) was much higher in juvenile than in adult DM patients (control vs juvenile DM: Mean ± SE: 0.06 ± 0.01 vs 0.6 ± 0.05; p < 0.0001 and control vs adult DM: Mean ± SE: 0.60 ± 0.1 vs 0.75 ± 0.1; p = 0.051). Gene expression analysis demonstrated that genes that participate not only in angiogenesis but also in leukocyte trafficking and the complement cascade were highly up regulated in DM muscle in comparison to age matched controls. DC-LAMP positive dendritic cells were highly enriched at perivascular inflammatory sites in juvenile and adult DM patients along with molecules that facilitate dendritic cell transmigration and reverse transmigration (CD142 and CD31).

**Conclusion:**

These results suggest active neovascularization and endothelial cell activation in both juvenile and adult DM. It is likely that close association of monocytes with endothelial cells initiate rapid dendritic cell maturation and an autoimmune response in DM.

## Background

Idiopathic inflammatory myopathies (polymyositis (PM); dermatomyositis (DM) and related conditions) are a heterogenous group of autoimmune disorders whose causes and pathogenesis remain unclear. In DM, inflammatory changes occur both in muscle and in skin. Although there has been no direct comparison, the pathological changes in juvenile and adult DM appear to be similar except that all the basic pathological features are more prominent in the childhood form. The main histopathologic alterations in DM are found in relation to the blood vessels of the connective tissue of the muscle, skin and gastrointestinal tract. In DM, the inflammatory exudate is predominantly perivascular and perimysial and to a lesser extent endomysial. In juvenile DM, intramuscular blood vessels also show endothelial hyperplasia, immune complex and complement deposition, and focal loss of capillaries [1]. It is believed that in adult DM, capillary loss precedes other pathological changes in the muscle, and that the capillary endothelium is an early and possibly primary target of immune attack [2,3]. A physiologic reaction to capillary loss would be formation of new blood vessels, but the evidence for such neovascularization in DM patients has not previously been investigated.

The recruitment of leukocytes involves sequential capture on, rolling along and firm adhesion to the microvascular endothelium, followed by transmigration of leukocytes through the vessel wall and further migration in extra-vascular tissue [4]. The steps in the recruitment cascade are orchestrated by the cell adhesion molecules on both leukocytes and endothelial cells; different subsets of cell adhesion molecules are responsible for different steps. Previous studies have shown that adhesion molecules which facilitate leukocyte transmigration are up-regulated in the capillaries of DM muscle tissue, suggesting an active participation in the recruitment of the inflammatory infiltrate into the muscle tissue [5]. Monocyte-derived dendritic cells play a critical role in controlling immunity by activating naïve T cells. Monocytes leave the blood stream by endothelial cell transmigration, engulf tissue antigens, differentiate into mature dendritic cells, and finally reverse-transmigrate into lymph nodes to activate naïve T-cells. Recent *in vitro *studies show that PECAM-1 (CD31) and tissue factor (CD142) play a critical role in transmigration and reverse transmigration respectively [6,7].

Angiogenesis is an important component of the inflammatory response, during which new vessels are formed from preexisting ones via sprouting and non sprouting mechanisms. Because the status of angiogenesis in myositis is not known, we have focused our attention on identifying the molecular processes that facilitate angiogenesis and the immune response in DM. Our first aim was to demonstrate whether there are signs of angiogenesis in muscle tissue of patients with juvenile and adult DM. A second aim was to identify molecular pathways that are relevant for angiogenesis by gene expression profiling using muscle biopsies from adult DM patients, and a third aim was to investigate whether dendritic cells and molecules that facilitate their entry and exit are present *in vivo *in the inflamed muscle tissue of DM patients.

## Methods

### Patients

All the human tissue samples were handled according to National Institutes of Health and Johns Hopkins School of Medicine IRB guidelines. Muscle biopsies from nine untreated juvenile DM patients (age, years: 6.7 ± 3.2) and six non-myositis childhood controls (age, years: 11.5 ± 2.9), along with six adult DM patients (age years: 39.4 ± 16.9) and seven normal adult controls (age, years: 37.5 ± 13.9) were used for immunohistochemistry. Myositis patients met diagnostic criteria of Bohan and Peter [8]. All adult DM patients had skin rash, muscle weakness, elevated serum levels of muscle enzymes and positive EMG or a positive muscle biopsy. Histological examination showed that inflammation was patchy and some muscle fibers looked apparently normal in some biopsies. It is not unusual for some muscle fibers in a DM biopsy to look apparently normal. Muscle biopsies from a separate group of 5 adult untreated female DM patients (age, years: 48.5 ± 14.8) were profiled and compared to muscle tissue of normal human muscle (NHM) from four healthy volunteers (age, years: 43.5 ± 12.1). Gene expression profiling was not done on Juvenile DM patients at this time due to the non availability of muscle tissue suitable for RNA extractions.

### Immunohistochemistry

Immunohistochemistry was performed as described previously [9]. The cell surface adhesion molecule CD146 has been identified as an endothelial cell marker [10], whereas the adhesion receptor αVβ3 has been identified as a marker of angiogenic vascular tissue [11]. We have used anti-CD146 antibodies as a pan-endothelial marker to assess all blood vessels and integrin αVβ3 antibodies to assess new blood vessel formation. The following primary antibodies were used to detect endothelial cell markers (mouse anti-human-αVβ3 (1:50) (Chemicon); mouse anti-human-CD146 (1:60) (Chemicon); anti-human CD31 (1:20) (Dako); anti-human CD142 (BD Pharmingen); and dendritic cell marker (mouse anti-human DC-LAMP (1:10) (Immunotech). Anti-mouse HRP (1:500) (Dako) and anti-rabbit HRP (1:500) (Dako) were used as secondary and tertiary antibodies. Isotype-matched mouse Igs and normal rabbit serum were used as negative controls. Muscle biopsies were serially sectioned (8–10 μm) and stained with CD146 (endothelial) and αVβ3 antibodies. Digital pictures (Axiovision V3.1 software) of stained sections were taken using a microscope (Axioscope, Carl Zeiss). Eight to ten non-overlapping fields (20X objective) per sample were used for image analysis. All samples were analyzed independently by two persons who were blinded to clinical status. The number of blood vessels or new capillaries per muscle fiber was calculated for each disease group. Statistical analysis was performed by Student's t-test.

### mRNA profiling

Total RNA was extracted from muscle biopsy or tissue individually using TRIzol Reagent (Life Technologies, Gaithersburg, MD). Ten micrograms of each total RNA sample were processed as previously described [12,13]. A single cRNA sample from each specimen was applied to Affymetrix U133A microarrays. Arrays were stained with phycoerythrin-streptavidin, and the signal intensity was amplified by treatment with a biotin-conjugated anti-streptavidin antibody followed by a second stain of phycoerythrin-streptavidin. Second-stained arrays were scanned on a Hewlett-Packard G2500A Gene Array Scanner with the photomultiplier tube set at 1800V.

### mRNA profile quality controls and normalization

The profiles were normalized for inter-chip intensity variation by scaling the overall intensity of each profile to 800, and absolute analysis for each profile was generated with Affymetrix Microarray Suite 5.0 software. Each profile was subjected to a stringent series of quality controls: scaling factor <4, present calls >30%, internal probe set controls 5'/3' ratios >0.6. All profiles passed quality control measures as described previously [14]. All files associated with this analysis are available on our web site [15]. All data is also deposited to NCBI GEO, via a custom automated submission pipeline between the Microarray Center at Children's National Medical Center and NCBI GEO. We note that there are a number of alternate methods for probe set analyses and normalizations for Affymetrix arrays (such as ProbeProfiler, dCHIP, RMA), and that different results can be obtained with different analysis methods. We have recently shown that an analysis using MAS 5.0, with a 10% present call filter provides excellent signal/noise levels for delineation of diagnostic groups in human muscle biopsies [16], and this is the method used for the current analysis.

### Data analysis

Gene-expression profiles were generated from disease and control groups using high-density oligonucleotide arrays. The MAS 5.0 and 10% present call filter was as in our previous publications [12,17,18]. U133A arrays have about 22,000 probe sets encoding mostly confirmed genes. Expression changes were initially assessed using Welch's two sample t test, without correction for multiple testing, using GeneSpring™ software (Silicon Genetics, Redwood City, CA). Differentially expressed genes between DM and NHM were identified by T-test (p < 0.05) and fold change (greater than 2 fold). Manually grouped genes were further clustered with GeneSpring to visualize clusters of genes with similar expression pattern. Gene tree was colored by the expression of average over the whole group. Gene function was based on information available in public databases or in the literature.

## Results

### Neoangiogenesis in DM

**Figure 1:**
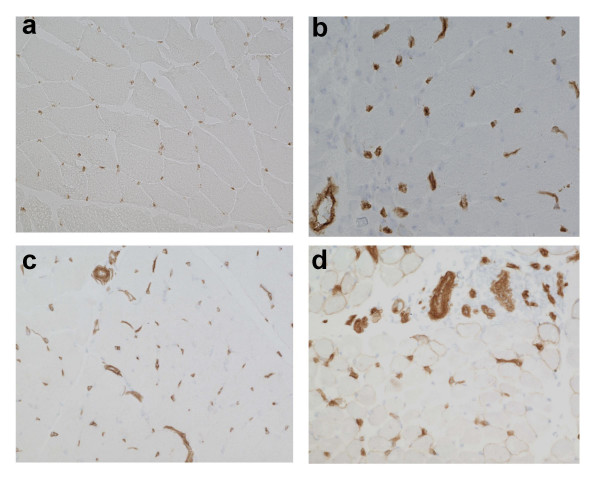
**Expression pattern of CD146 in juvenile dermatomyositis (DM), adult DM, and control muscle biopsies**. Frozen muscle sections from myositis and control samples were stained with antibodies that recognize pan endothelial marker CD146. Representative staining patterns for CD146 in normal human muscle control (panels a&c) and DM (panel b) and juvenile DM (panel d). Note strong endothelial staining in the capillaries of both DM and juvenile DM patients.

**Figure 2:**
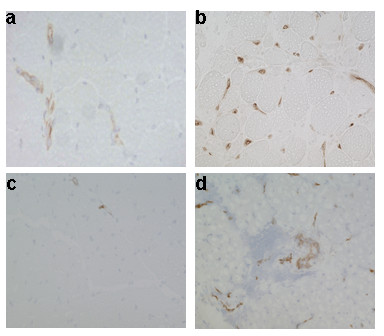
**Expression pattern of αVβ3 in juvenile DM, adult DM, and control muscle biopsies**. Frozen muscle sections from myositis and normal human muscle control samples were stained with antibodies that recognize αVβ3. Representative staining patterns for αVβ3 in control (panels a&c) and DM (panel b) and juvenile DM (panel d).

Blood vessels in normal muscle tissues weakly stained for CD146, whereas a more intense staining was observed in capillaries of (endothelial cells and pericytes) adult and juvenile DM biopsies (Fig. 1). We also have observed some CD146 non-specific staining on some muscle fibers of patient biopsies. Capillaries in normal muscle tissues were generally negative for the neoangiogenesis marker αVβ3 except for occasionally positive large vessels, whereas a significant number of capillaries and large blood vessels were strongly stained for αVβ3 in the adult and juvenile DM biopsies (Fig. 2). Estimation of the number of CD146 positive blood vessels (large vessels and capillaries) per muscle fiber showed no significant differences between juvenile DM and childhood controls (control vs JDM: Mean ± SE: 1.0 ± 0.2 vs 1.0 ± 0.3; p = ns); whereas the number of CD146 positive blood vessels per fiber was significantly reduced in adult DM as compared to adult controls (control vs DM: Mean ± SE: 2.2 ± 0.3 vs 1.3 ± 0.2; p < 0.05). Estimation of the number of αVβ3 positive blood vessels showed significantly higher number of αVβ3 positive blood vessels per fiber in the JDM biopsies compared to the childhood controls (control vs JDM: Mean ± SE: 0.06 ± 0.01 vs 0.6 ± 0.05; p < 0.0001). In the biopsies from the adult DM patients there was also an increase in αVβ3 positive blood vessels per fiber in comparison to adult controls, but this was not significant (control vs DM: Mean ± SE: 0.60 ± 0.1 vs 0.75 ± 0.1; p = 0.051).

### Angiogenic and anti-angiogenic genes in DM

**Figure 3:**
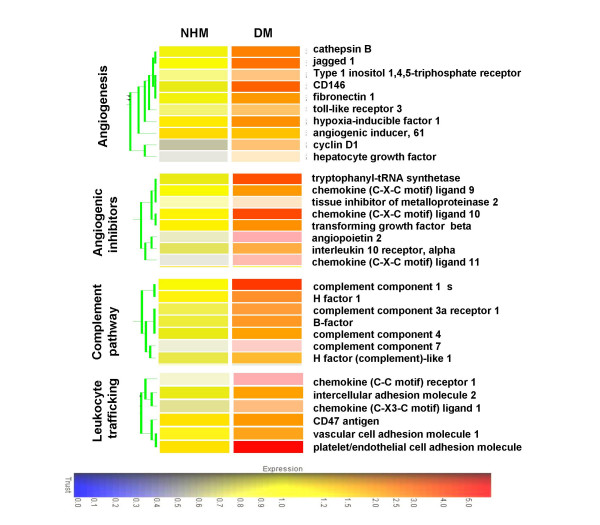
**Members of the angiogenic, anti-angiogenic, lymphocyte trafficking and complement activation pathways are highly up-regulated in DM patients compared to normal human muscle (NHM) controls**. Gene-expression profiles generated from disease and control groups were analyzed using high-density oligonucleotide arrays. Gene expression values were obtained following Affymetrix MAS5 normalization. Manually grouped genes were further clustered with GeneSpring to visualize clusters of genes with similar expression pattern. Gene tree was colored by the expression of average over the whole group. Gene function was based on information available in public databases or in the literature.

Because of the evidence of increased angiogenesis in DM, we next decided to look at the status of genes that influence the angiogenic pathway by global gene expression profiling. Gene expression profile analysis of biopsies from adult DM patients showed that mRNA levels of the genes that participate in endothelial adhesion (e.g., cathepsin B (CTSB), Endo 1-associated antigen (CD146)), proliferation (e.g., cyclin D1), differentiation (e.g., jagged protein), migration (e.g., hepatocyte growth factor), and activation (e.g., inositol 1,4,5 trisphosphate receptor type 1 (ITPR1), hypoxia-inducible factor 1 alpha subunit (HIF1A), toll-like receptor 3, and angiogenic inducer 61) are up-regulated in DM patients compared to controls. This suggests that critical molecules required for initiation of angiogenic response-endothelial cell migration, and proliferation and maturation of neovasculature, are active in the DM patients in comparison to controls (Fig. 3).

Whether or not angiogenesis occurs in a particular tissue depends on the balance between the relative amounts of pro- and anti-angiogenic factors [19]. Analysis of myositis and control muscle tissues also showed a significant up regulation of anti-angiogenic genes (angiopoietin-2, tryptophenyl-tRNA synthetase, interleukin 10 receptor, and TGF-beta) in DM patients suggesting an active ongoing anti-angiogenic response (Fig. 3).

The endothelial markers originally identified to be associated with leukocyte recruitment are also involved in neovascularization, suggesting a dual role not only in leukocyte-endothelial adhesion but also in angiogenesis [20]. Significant up-regulation of the markers that participate in both leukocyte trafficking and angiogenesis (e.g., CX3CL1, CCR1, CD47, VCAM-1, ICAM-1, PECAM1 and ICAM2) is noted in DM patients (Fig. 3).

It has been previously shown that immune complex and complement deposition occurs in arterioles and capillaries of DM patients [2,21]. Since the complement cascade is intricately associated not only with vessel damage but also with angiogenesis, we have specifically looked for members of the complement cascade that facilitate angiogenesis. We found that both classical and alternate complement cascade members along with regulators (Factor-B, C7, Factor-I, C1S, Factor H, and C4) are highly up regulated in DM patients (Fig. 3).

### DC-LAMP positive dendritic cells in DM

**Figure 4:**
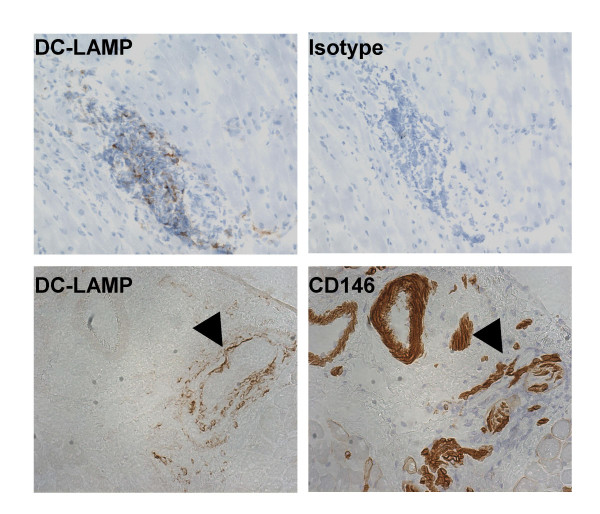
**DC-LAMP positive dendritic cells are enriched in perivascular area of inflammation in juvenile DM patients**. Juvenile DM and control biopsies were stained with antibodies that recognize dendritic cell marker DC-LAMP along with an isotype matched control (upper panels). Consecutive sections stained with DC-LAMP and CD146 showing close proximity of DC-LAMP positive cells to blood vessels. Normal biopsies showed no staining with DC-LAMP antibodies (data not shown).

**Figure 5:**
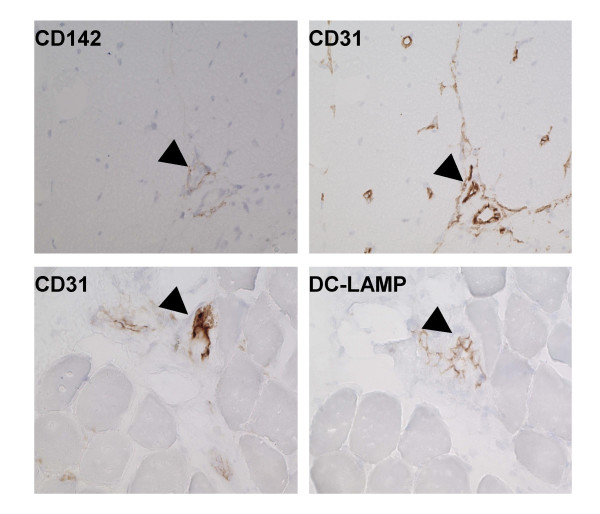
**Expression pattern of CD142, CD31 and DC-LAMP in juvenile DM patients**. Serial sections of juvenile DM biopsies stained with anti-tissue factor (CD142) and PECAM1 (CD31) antibodies (upper panel). Serial sections stained with CD31 and DC-LAMP showing close proximity of DC-LAMP positive cells to CD31 positive blood vessels (arrows).

Gene expression analysis of adult DM samples clearly showed that both PECAM1 and tissue factor are highly upregulated in these patients as are several dendritic cell/macrophage maturation markers (FLT3 ligand, CD68, CD11B, DC-LAMP, HLA-DQ, HLA-DRB, and B7-2) (data not shown). We have confirmed the presence of DC-LAMP positive dendritic cells at the perivascular inflammatory sites, often in close proximity to blood vessels in myositis patients (Fig. 4). Immunohistochemical analysis also suggests that the genes that facilitate the transmigration and reverse transmigration (CD31 and CD142) of dendritic cells are upregulated in blood vessels of both juvenile (Fig. 5 upper panels) and adult DM patients (data not shown) and DC-LAMP positive dendritic cells in close proximity to CD31 positive blood vessels (Fig. 5 lower panels). We found modest blood vessel staining for both CD 31 and CD 142 in control biopsies (data not shown).

## Discussion

Immunohistochemical and global gene expression analysis of independent groups of DM muscle samples clearly showed that angiogenesis and genes that influence angiogenesis, leukocyte migration, and complement activation are highly upregulated in DM. Expression profiling also suggested that several dendritic cell markers are up-regulated in DM patients. We have confirmed the presence of DC-LAMP positive dendritic cells that are predominantly localized in perivascular areas of inflammation and in close proximity to blood vessels. Juvenile and adult DM patients also had dendritic cells in close proximity to blood vessels expressing antigens (e.g., PECAM and Tissue factor) that facilitate their transmigration and reverse transmigration.

It is known that skeletal muscle capillarity is very dynamic, for example repeated exercise results in increased capillarity and inactivity results in reduced capillarity [22]. There is little information, however, regarding the molecular pathways that influence capillary formation in inflammatory muscle diseases. Angiogenesis is generally quiescent in adults with the exception of certain tightly controlled physiological situations such as female reproductive functions and tissue regeneration and repair [23]. It is widely accepted that the "angiogenic switch" is 'off' when the effect of pro-angiogenic molecules is balanced by that of anti-angiogenic molecules and is 'on' when the net balance is tipped in favor of angiogenesis [24]. The relative contributions of both pro- and anti-angiogenic factors are likely to change the vascular network in a diseased tissue. Although the exact mechanisms controlling angiogenesis in inflamed muscle are not fully understood, mechanistically, the major stimuli for neo-vascularization are hypoxia and inflammation. Inflammatory signals recruit lymphocytes and macrophages into areas of neovascularization which further act as a source of angiogenic and arteriogenic factors.

It has been previously shown that there is significant capillary loss in the muscle of adult DM patients associated with deposition of the late components of the complement pathway [21]. Our current findings using CD 146 antibodies support and confirm the previous results in adult DM. It was also suggested that similar loss of capillaries occur in juvenile DM patients, but there have been no systematic quantitative studies addressing the capillary loss in juvenile DM patients. Our data in juvenile DM patients in comparison with childhood non-inflammatory myopathic controls suggest that there is no significant net capillary loss despite activated complement system in juvenile DM patients. These results suggest that either capillary loss is effectively compensated by robust new capillary formation or complement mediated damage is modest. In adult DM the number of αVβ3 positive (new) capillaries is not significantly different from that of controls. The modest increase in new capillaries in adult DM also suggests ongoing loss of existing capillaries. Our findings further confirm and extend previously published gene expression data as well as the recent studies showing up-regulation of angiogenesis-related factors (HIF-1beta, alphaV beta3, VEGFR-1) in DM biopsies [25,26]

Although previous work has shown a decrease in capillaries in DM but not PM, our results suggest that there is a significant upregulation of several pathways that initiate angiogenesis in myositis. The new capillaries are highly significantly increased in JDM in comparison with respective controls. Thus it appears that the association between myositis lesions and neovascularization is not merely an epiphenomenon, but represents a compensatory mechanism. In this study all adult DM patients were used for immunohistochemical analysis were treated with Prednisone. The differences in angiogenic responses between juvenile and adult DM patients are likely not due to the medication, because gene expression profiling of untreated adult DM patients also show up-regulated angiogenic genes.

Pathological conditions, such as the inflammatory process, may perturb the resting state of the endothelial cells and promote transendothelial migration of cells and leakage of molecules that are needed at specific sites. Leukocyte interactions with inflamed endothelial cells are mediated by selectins, signaling molecules that include lipids and chemokines, integrins and their ligands, and junctional molecules. Several of these molecules that were initially identified to have a role in leukocyte trafficking have also been found to influence endothelial cell activation, proliferation and differentiation. For example, different complement activation products can perturb the antithrombotic state of quiescent endothelial cells by various mechanisms for example C5a causes the release of heparin sulfate from endothelial cells, and the membrane attack complex is now widely recognized as a potent promoter of coagulation [27]. The membrane attack complex added to endothelial cells in sublytic concentrations induces release of von Willebrand Factor, which in turn favors platelet adherence to the vessel wall [28], and promotes the assembly of prothrombinase by the exposure of phospholipids [29].

Dendritic cells are antigen presenting cells with the unique ability to initiate an immune response. Immature dendritic cells are localized in peripheral tissues where they exert a sentinel function for incoming antigens. After antigen capture and exposure to inflammatory stimuli, dendritic cells undergo maturation and migrate to regional lymph nodes where the presentation of antigenic peptides to T lymphocytes takes place. It is known that some monocytes differentiate into dendritic cells in response to cues that are endogenous to endothelial cells [6,30]. Passage of leukocytes across the endothelial lining into sites of inflammation has been shown to be regulated largely by platelet/endothelial cell adhesion molecule-1 (PECAM/CD31) and the reverse transmigration involves p-glycoprotein and tissue factor expressed on the leukocytes. The reverse transmigrating cells were shown to be dendritic cells [6]. Both tissue factor and PECAM are highly significantly expressed DM patients suggesting an active participation in dendritic cell migration.

## Conclusion

In summary, these results suggest that neoangiogenesis is highly activated in DM patients. It appears that significant new blood vessel formation in patients is influenced not only by traditional pro-and anti-angiogenic genes, but also by genes that participate in leukocyte trafficking and complement activation pathways. Further it is likely that close association of monocytes with activated endothelial cells will initiate rapid dendritic cell maturation and initiation of autoimmune response in DM.

## Competing interests

The author(s) declare that they have no competing interests.

## Authors' contributions

KN, RR, MM, CF, YC conducted research, KN, LGR, FW, PHP, KP, EHP, IL, provided samples, designed research, analyzed data and wrote paper. KN oversaw research, designed and conducted experiments, analyzed data, and wrote paper.
